# Associations between common diseases and work ability and sick leave among health care workers

**DOI:** 10.1007/s00420-017-1231-1

**Published:** 2017-05-26

**Authors:** Sophie van den Berg, Alex Burdorf, Suzan J.W. Robroek

**Affiliations:** Department of Public Health, Erasmus Medical Center Rotterdam, PO Box 2040, 3000 CA Rotterdam, The Netherlands

**Keywords:** Sick leave, Health behavior, Psychosocial work demands, Physical work demands, Health care

## Abstract

**Purpose:**

This study investigates whether common diseases, i.e., musculoskeletal diseases (MSD), cardiovascular diseases (CVD), mental disorders (MD), and respiratory diseases (RD), influence work ability and sick leave and whether lifestyle-related factors, and psychosocial and physical work-related factors are associated with low work ability and sick leave.

**Methods:**

In a cross-sectional study among 8364 Dutch health care employees, self-reported information was acquired concerning common diseases, lifestyle-related factors, psychosocial and physical work-related factors, work ability, and sick leave. Logistic regression analyses were performed to describe the associations between common diseases with low work ability and sick leave, and to evaluate differences in associations between lifestyle-related and work-related factors with low work ability and sick leave among healthy employees and employees with common diseases.

**Results:**

Employees with MD (OR 6.35), CVD (OR 2.63), MSD (OR 2.62), and RD (OR 2.11) had a higher risk of low work ability compared to healthy employees. Workers with common diseases also reported more often sick leave (ORs > 1.60), in particular long-term sick leave (>25 days). Multimorbidity increased both the occurrence of low work ability and sick leave. Unfavourable psychosocial work-related factors were associated with low work ability and sick leave regardless of health status. Physical work-related factors and lifestyle factors were less consistently associated with low work ability and sick leave.

**Conclusions:**

Common diseases, and foremost mental disorders, were related to both low work ability and sick leave. To maintain or improve work ability and prevent sick leave, interventions that promote a healthy psychosocial work environment are needed.

## Background

There is a need for employees to remain productive until retirement age. This need is particularly apparent in health care, due to a shortage of nurses and a high turnover of nursing personnel (Hayes et al. 2006). Ageing employees have an increased risk of health problems and several studies have shown that poor self-rated health can lead to productivity loss at work, low work ability, sick leave and early exit from paid employment (Alavinia et al. 2009; Laaksonen et al. 2011; Van Rijn et al. 2014; Van de Vijfeijke et al. 2013). However, less is known about the impact of specific common diseases on sick leave and work ability. Furthermore, knowledge on the role of modifiable lifestyle-related and work-related factors is essential for designing effective interventions to maintain or improve the work ability of workers with health problems and to prevent long-term sickness absence.

In the European Union, 46% of people within the labour market reported to have work limitations due to health problems (Eurostat 2015). Within the health care sector, a particular large proportion of over 60% of the employees was found to have health problems (Godderis et al. 2015). Musculoskeletal diseases (MSD), cardiovascular diseases (CVD), mental disorders (MD), and respiratory diseases (RD) have been identified as important causes of sick leave (Ferrie et al. 2009; Vahtera et al. 2010). A recent longitudinal study showed associations between individuals with MSD, CVD, and MD and decreased work ability (Leijten et al. 2014). Particularly, employees with psychological problems seem to be at risk for low work ability, productivity loss at work, and sick leave compared to employees with other common diseases (Leijten et al. 2013, 2014; Van den Heuvel et al. 2010). As regards comorbidity, with increased number of chronic diseases the likelihood of sick leave increased (Ward 2015).

Several studies have shown that demographics, particularly older age and lower educational level, lifestyle-related characteristics, particularly obesity, smoking, and lack of leisure time physical activity, as well as physical and psychosocial work-related factors are associated with poor work ability and sick leave (Robroek et al. 2011, 2013; Van den Berg et al. 2009). It is estimated that 10% of sick leave may be attributed to lifestyle behaviours and obesity (Robroek et al. 2011). Less well studied is the role of lifestyle-related and work-related factors on work ability and sick leave in employees with common diseases. A longitudinal study reported that lower autonomy increased the likelihood of long-term sick leave among employees with MSD, CVD, and psychological problems, while high job demands increased the likelihood of sick leave among employees with psychological complaints only (Leijten et al. 2013). Gaining more insight into determinants of low work ability and sickness absence among employees with common diseases provides information whether generic or disease-specific interventions should be applied regarding lifestyle- and work-related factors.

The objective of this study is to investigate whether common diseases, i.e., musculoskeletal diseases (MSD), cardiovascular diseases (CVD), mental disorders (MD), and respiratory diseases (RD), influence work ability and sick leave and whether lifestyle-related factors, and psychosocial and physical work-related factors are associated with low work ability and sick leave.

## Methods

### Study design

The study population of this cross-sectional study consisted of employees from 18 health care organizations in Limburg, The Netherlands. The health care organizations include home care organizations, nursing homes, mental health care organizations, homes for physically or mentally handicapped persons, a rehabilitation centre, a maternal care organization, and a hospital. The organizations implemented, in collaboration with an occupational health organization, a programme to get insight into the sustainable employability of their employees. In 2011/2012, the employees were invited to fill in an online questionnaire with questions on lifestyle, work, and health. At the start, all invited employees were informed that the information was also used for scientific purposes and that filling in the questionnaire was interpreted as informed consent. All data were anonymized for privacy protection (Reeuwijk et al. 2014). In total, 8426 of the 15,358 invited employees responded (55%). Reasons for non-participation are unknown. In total, 62 employees were excluded because of incomplete data (*n* = 37) and because of suspected incorrect response (*n* = 25), as they filled out on the questionnaire to have all the 14 disease categories which was considered unlikely—resulting in a study population of 8364 employees.

### Measures

#### Work ability score

To assess work ability, a single item question of the work ability index was used (the work ability score, WAS) (Gould et al. 2008) in which employees were asked to rate their current work ability relative to the best work ability during their life on a scale from 0 (unable to work) to 10 (current work ability equals best work ability ever). A low work ability was defined as a score of 7 or lower.

#### Sick leave

Employees were asked to indicate on a 5-point scale how many days of sick leave that they had during the past year. Sick leave was dichotomized into 0 day sick leave and 1–365 day sick leave. For further exploration, the categories were further divided into four categories (0 days, 1–9 days, 10–24 days or 25–365 days) (Robroek et al. 2011).

#### Common diseases

The presence of diseases was also assessed using the work ability index. Participants were asked to indicate on a list of 13 broad disease categories (i.e., accident, MSD, CVD, RD, MD, neurological disorders, digestive disorders, genitourinary disorders, skin disorders, tumours, endocrine disorders, blood disorders, heritable disorders, and others) which of the diseases were currently present as diagnosed by a physician (yes/no). Employees were regarded healthy when none of these diseases were present. This question was used to select workers with MSD, CVD, RD, and MD. Multimorbidity was defined as having two or more of these selected diseases.

#### Lifestyle-related factors

Excessive alcohol intake, heavy smoking, and leisure time physical activity (PA) were assessed with single yes/no questions. Excessive alcohol intake was defined as having more than 10 alcoholic beverages a week, and heavy smoking as having more than 20 cigarettes a day. Insufficient leisure time physical activity was defined as having less than 30 min of PA or sports participation during leisure time a day (Robroek et al. 2011). Body mass index (BMI) was calculated based on self-reported height and weight, and classified as normal weight (BMI ≤ 25 kg/m^2^), overweight (25–29 kg/m^2^), and obesity (BMI ≥ 30 kg/m^2^).

#### Physical work-related factors

The physical work-related risk factors included lifting heavy loads of more than 25 kg, working in an awkward posture and working in a static posture. The physical work-related factors were assessed with single questions and were rated by the participant as never, sometimes, often or always. For this study employees were classified into those with a low physical workload (never, sometimes) and those with high physical workload (often and always) (Elders and Burdorf 2001).

#### Psychosocial work-related factors

Self-reported work-related factors included work demands, job control, and perceived rewards. Employees rated their work demands on five questions concerning excessive work, working fast, and time pressure (Cronbach’s alpha = 0.86) with answers ranging from ‘never’ (1) to ‘always’ (4). The five items on job control assessed whether the respondent can influence the planning of tasks, pace of work, decisions about carrying out the tasks, interruption of work, and deadlines (Cronbach’s alpha = 0.81), with answers ranging from ‘never’ (1) to ‘always’ (4). For both work demands and job control, a sum score was calculated, and employees in, respectively, the upper and lowest quartile were considered to be exposed to higher work demands and lower job control.

Perceived rewards were assessed using a single question asking whether the organization provides the rewards which he or she deserves for the work. Those answering the question with ‘agree’ or ‘totally agree’ were considered to have high self-reported rewards, while those answering ‘disagree’ or ‘totally disagree’ were considered to have low rewards.

#### Demographics

Information on gender, age, and education was collected. Age was divided into four groups: less than 30 years, 30–39 years, 40–49 years, and 50 years or older. Education was categorized into low (primary school, intermediate secondary, and lower vocational school), intermediate (higher secondary school and intermediate vocational schooling), and high (higher vocational schooling and university).

### Statistical analysis

Descriptive statistics (frequencies and percentages) were generated to report on characteristics of the study population. Logistic regression analyses were performed to analyse the associations between common diseases as independent variables with low work ability and sick leave as dependent variables. Similarly, the associations between lifestyle-related factors and work-related factors with the dependent variables, low work ability, and sick leave were estimated, stratified by healthy employees and employees with common diseases. Gender, age, and education level were included as potential confounders in all analyses, since they have been shown to be associated with work-related factors and both work ability and sick leave. All analyses were carried out with the Statistical Package for Social Sciences version 21.0 for windows.

## Results

The mean age of the study population consisting of 8364 Dutch health care employees was 49 years (SD ± 11 years). The majority of the participating employees was female (82%), and most employees completed intermediate (51%) or high (38%) education. Low work ability was present in 29.4%, and 54.3% was at least one day off work during the past year due to sick leave. Of the participating health care employees 39.1% had MSD, 12.7% CVD, 11.6% RD, and 7.9% MD (Table 1). Unhealthy lifestyle-related factors and unfavourable physical and psychosocial work-related factors were more prevalent among employees with common diseases than among healthy employees.

**Table 1:** Demographic, lifestyle, and work-related characteristics among healthy workers and among workers with musculoskeletal disease (MSD), cardiovascular disease (CVD), mental disorders (MD), and respiratory disease (RD)

	Healthy workers *n* = 2858 (34.2%)	Workers with MSD *n* = 3271 (39.1%)	Workers with CVD *n* = 1066 (12.7%)	Workers with MD *n* = 661 (7.9%)	Workers with RD *n* = 969 (11.6%)
	%	%	%	%	%
Demographics					
Female gender	81.2	83.2	74.8	80.8	81.0
Age, <30 years	20.1	12.2	3.5	14.1	19.0
Age, 30–39 years	23.3	16.4	7.6	20.0	20.4
Age, 40–49 years	27.9	28.3	25.2	27.6	26.8
Age, ≥50 years	28.7	43.1	63.7	38.3	33.7
Education, low	10.1	13.6	17.6	12.3	10.4
Education, intermediate	45.9	55.9	49.4	56.1	54.5
Education, high	43.9	30.5	32.9	31.3	35.1
Lifestyle-related factors					
Heavy smoking	2.7	3.7	3.1	8.1	3.8
>10 glasses alcohol/wk	8.6	9.0	9.6	9.0	9.3
<30 min/day PA	30.3	33.2	32.6	36.0	36.3
BMI, normal weight	65.1	51.1	38.0	53.6	49.6
BMI, overweight	28.1	35.1	40.4	31.3	34.3
BMI, obese	6.9	13.8	21.6	15.8	16.1
Physical work-related factors					
Lifting heavy loads	7.8	12.2	9.3	13.3	11.2
Awkward posture	12.0	22.4	20.1	23.4	21.4
Static posture	22.6	29.7	30.1	29.0	27.7
Psychosocial work-related factors					
Higher work demands	21.6	28.7	30.4	41.6	27.8
Lower job control	25.3	29.7	29.2	38.0	31.7
Lower rewards	26.9	35.0	37.9	47.2	36.1
Work-related outcomes					
Low work ability	17.8	38.7	38.5	62.5	34.6
Sick leave 0 days	56.1	38.7	40.8	23.6	34.9
Sick leave 1–9 days	37.1	38.5	37.2	38.0	44.1
Sick leave 10–24 days	4.1	9.5	9.1	11.8	9.8
Sick leave 25–365 days	0.6	13.3	12.9	26.6	11.2

In total, 34.2% had no disease and were considered healthy, 36.7% had one of the common diseases, 12.5% two, and 2.8% had three or all common diseases. Comparable with the total study population, respectively, 37, 36, and 33% of the individuals with CVD, MD, and RD also had MSD. Work ability and sick leave were weakly correlated (Spearman’s rho 0.16).

Employees with MSD, CVD, MD, and RD were more likely to have a low work ability compared to healthy employees after adjustment for demographics, and lifestyle-related and work-related factors. The ORs for low work ability for employees with MSD were 2.62 (95% CI 2.31–2.97), for employees with CVD 2.63 (95% CI 2.18–3.16), MD 6.35 (95% CI 5.21–7.73), and RD 2.11 (95% CI 1.77–2.51) compared to healthy participants.

**Fig. 1:**
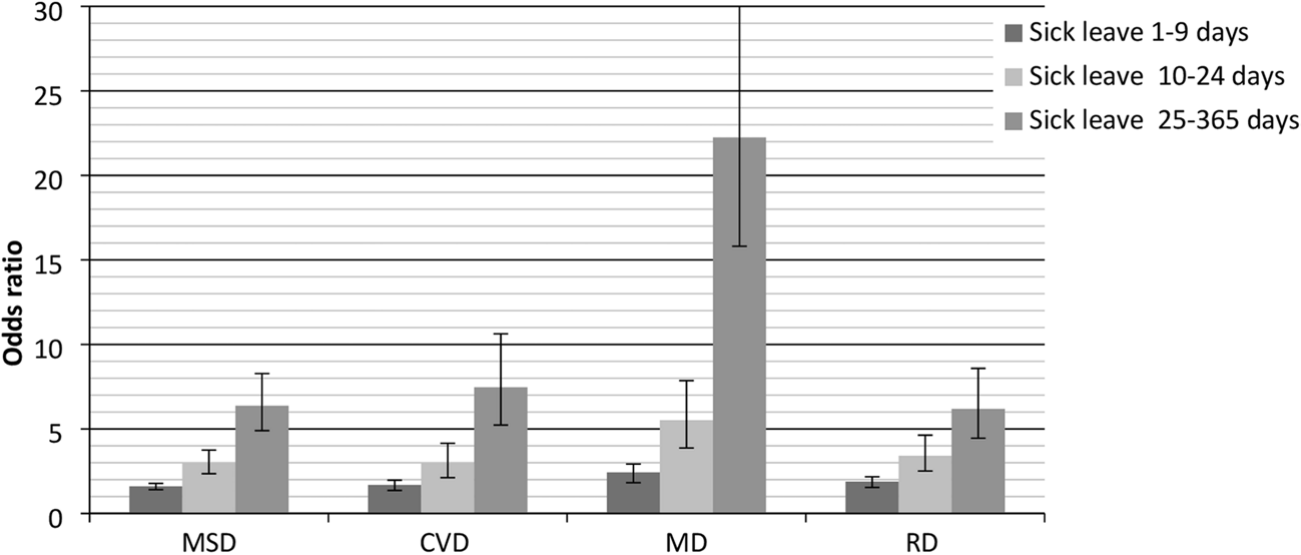
Associations between MSD, CVD, MD, RD, and sickness absence, after adjustment for demographics, lifestyle-related factors, and work-related factors as compared to healthy employees

Employees with MSD, CVD, MD, and RD were also more likely to have at least 1 day of sick leave, and in particular long-term sick leave (Fig. 1). The ORs for 25–365 days of sick leave were 6.37 (95% CI 4.90–8.28) for employees with MSD, 7.46 (95% CI 5.23–10.63) for employees with CVD, 22.24 (95% CI 15.81–31.30) for employees with MD, and 6.18 (95% CI 4.45–8.58) for employees with RD.

Employees with one of these common diseases were 2.13 times (95% CI 1.88–2.42) more likely to have low work ability, employees with two diseases 3.41 times (95% CI 2.8–4.04), and employees with three or four diseases 5.43 times (95% CI 3.50–6.64).

**Fig. 2:**
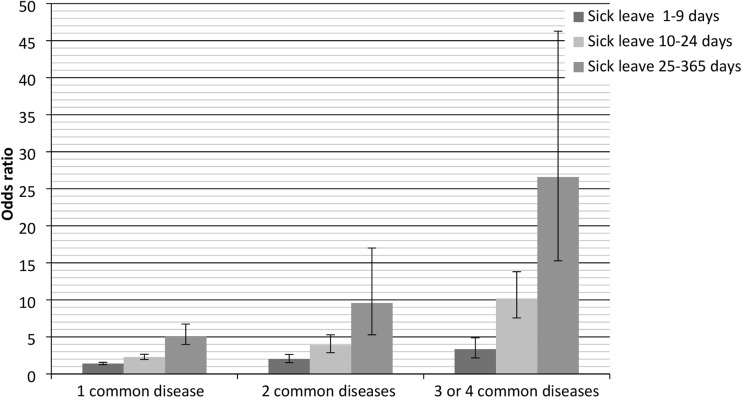
Associations between sick leave and employees with multiple common diseases adjusted for demographics, lifestyle-related factors, and work-related factors as compared to healthy employees

Employees with two or more diseases were also more likely to be off work due to sick leave, particularly long-term sick leave (Fig. 2). For employees with MSD, the OR for 25–365 days of sick leave was 5.02 (95% CI 3.84–6.55); for employees with two diseases 9.58 (95% CI 6.95–13.21); and for employees with three or four diseases 26.59 (95% CI 15.27–46.28). Within the subgroups of common diseases, comorbidity was strongly associated with low work ability and sick leave, except for employees with MD (data not shown).

Within healthy employees as well as within the subgroups with common diseases—except for MD—unfavourable physical work-related factors were associated with low work ability (Table 2). Psychosocial work demands were statistically significantly associated with low work ability among all employees, regardless of the presence of a common disease, with ORs ranging from 1.40 (95% CI 1.00–1.96) for low job control in employees with MD to 2.88 (95% CI 2.33–3.54) for high work demands in healthy employees. High work demands had, on average, the strongest association with low work ability, followed by low rewards and low job control. Regarding lifestyle factors, a low work ability was more prevalent among healthy workers with insufficient PA, and, although not statistically significant, among workers with MSD and insufficient PA. Other lifestyle-related factors, namely heavy smoking and obesity, were only statistically significantly related to low work ability in the subgroup of workers with RD.

**Table 2:** Associations between unfavourable lifestyle-related and work-related factors and low work ability score among healthy workers (*n* = 2858) and workers with MSD (*n* = 3271), CVD (*n* = 1066), MD (*n* = 661), and RD (*n* = 969)

	Low work ability				
	Healthy workers *n* = 509 (18%)	Workers with MSD *n* = 1267 (39%)	Workers with CVD *n* = 410 (38%)	Workers with MD *n* = 413 (62%)	Workers with RD *n* = 335 (35%)
	OR (95%CI)	OR (95% CI)	OR (95% CI)	OR (95% CI)	OR (95% CI)
Lifestyle-related factors					
Heavy smoking	1.15 (0.65–2.04)	1.02 (0.70–1.49)	1.26 (0.63–2.55)	0.92 (0.52–1.65)	2.32 (1.20–4.48)^*^
>10 glasses alcohol/wk	1.23 (0.87–1.74)	1.28 (0.99–1.65)	1.00 (0.64–1.54)	1.18 (0.66–2.11)	1.52 (0.96– 2.41)
<30 min/day PA	1.49 (1.22–1.82)*	1.44 (0.98–1.33)	1.10 (0.84–1.44)	1.00 (0.71–1.39)	1.20 (0.91–1.58)
BMI overweight	0.97 (0.77–1.20)	0.88 (0.75–1.03)	0.94 (0.71–1.25)	0.81 (0.56–1.16)	1.03 (0.76–1.39)
BMI obese	0.95 (0.64–1.39)	1.13 (0.91–1.40)	1.00 (0.71–1.39)	0.82 (0.52–1.30)	1.59 (1.09–2.32)*
Physical work-related factors					
Lifting heavy loads	1.13 (0.80–1.61)	1.38 (1.11–1.71)*	1.52 (1.00–2.32)*	0.90 (0.55–1.42)	1.52 (1.01–2.29)*
Awkward posture	1.72 (1.32–2.25)*	1.92 (1.62–2.27)*	2.72 (1.98–3.73)*	1.06 (0.72–1.56)	1.79 (1.30–2.47)*
Static posture	1.24 (0.99–1.55)	1.19 (1.02–1.39)*	1.39 (1.06–1.82)*	0.94 (0.66–1.33)	1.48 (1.10–1.99)*
Psychosocial work-related factors					
Higher work demands	2.88 (2.33–3.54)*	2.13 (1.82–2.50)*	2.54 (1.93–3.33)*	1.64 (1.18–2.27)*	2.50 (1.86–3.36)*
Lower job control	1.73 (1.40–2.13)*	1.62 (1.39–1.89)*	1.76 (1.33–2.32)*	1.40 (1.00–1.96)*	1.78 (1.33–2.37)*
Lower rewards	2.32 (1.90–2.84)*	2.13 (1.84–2.48)*	2.29 (1.76–2.97)*	1.82 (1.31–2.51)*	1.84 (1.39–2.42)*

Adjusted for gender, age, and educational level

*OR* odds ratio*, 95% CI* 95% confidence interval, *p < *0.05*

Compared to low work ability, unfavourable psychosocial work-related factors were to a smaller extent associated with the presence of sick leave (Table 3). Among employees who reported to receive low rewards, sick leave was more prevalent (ORs ranging between 1.12 and 1.33). Low job control was statistically significantly associated with sick leave among employees with MSD and CVD. High work demands were only statistically significantly associated with sick leave in healthy employees. High physical work demands (ORs ranging between 1.05 and 2.60) were associated with sick leave as well. Regarding lifestyle factors, heavy smoking was most strongly associated with sick leave among employees with CVD (OR 3.39, 95% CI 1.38–8.32) and MD (OR 2.86 95%, CI 1.11–7.38), and sick leave was more prevalent among individuals with insufficient physical activity and obesity. These associations were only statistically significant among healthy individuals (OR 1.23, 95% CI 1.05–1.45 for insufficient physical activity) and workers with MSD (OR 1.37, 95% CI 1.10–1.71 for obesity). Regarding heavy alcohol intake, inconsistent findings were found, with a lower risk of sick leave for employees with MD.

**Table 3:** Associations between unfavourable lifestyle-related and work-related factors sick leave among healthy workers (*n* = 2858) and workers with MSD (*n* = 3271), CVD (*n* = 1066), MD (*n* = 661), and RD (*n* = 969)

	Sick leave 1–365 days				
	Healthy workers *n* = 1255 (44%)	Workers with MSD *n* = 2005 (61%)	Workers with CVD *n* = 631 (59%)	Workers with MD *n* = 505 (76%)	Workers with RD *n* = 631 (65%)
	OR (95% CI)	OR (95% CI)	OR (95% CI)	OR (95% CI)	OR (95% CI)
Lifestyle-related factors					
Heavy smoking	1.23 (0.78–1.95)	1.24 (0.84–1.82)	3.39 (1.38–8.32)*	2.86 (1.11–7.38)*	1.24 (0.61–2.50)
>10 glasses alcohol/wk	1.10 (0.84–1.45)	1.02 (0.79–1.32)*	0.80 (0.52–1.23)	0.44 (0.24–0.78)*	0.96 (0.60–1.53)
<30 min/day PA	1.23 (1.05–1.45)	1.08 (0.93–1.26)	1.06 (0.81–1.37)	1.18 (0.80–1.72)	1.12 (0.85–1.48)
BMI overweight	1.16 (0.94–1.32)	1.00 (0.86–1.17)	1.09 (0.82–1.44)	0.78 (0.52–1.18)	1.09 (0.81–1.47)
BMI obese	1.04 (0.77–1.40)	1.37 (1.10–1.71)*	1.11 (0.80–1.55)	1.37 (0.76–2.46)	1.35 (0.91–2.00)
Physical work–related factors					
Lifting heavy loads	1.09 (0.82–1.44)	1.05 (0.76–1.02)	1.55 (0.99–2.44)	1.58 (0.85–2.94)	1.15 (0.81–1.63)
Awkward working posture	1.06 (0.84–1.34)	1.29 (1.08–1.54)*	1.41 (1.02–1.95)*	2.60 (1.51–4.46)*	1.14 (0.81–1.62)
Static working posture	1.26 (1.06–1.50)*	1.11 (0.95–1.30)	1.06 (0.81–1.38)	1.36 (0.90–2.07)	1.51 (1.11–2.06)*
Psychosocial work-related factors					
Higher work demands	1.28 (1.06–1.53)*	1.06 (0.90–1.24)	1.22 (0.93–1.60)	1.08 (0.75–1.57)	0.92 (0.68–1.24)
Lower job control	0.93 (0.78–1.11)	1.19 (1.01–1.39)*	1.37 (1.04–1.82)*	1.23 (0.83–1.82)	1.05 (0.78–1.40)
Lower rewards	1.23 (1.04–1.46)*	1.12 (0.97–1.30)	1.24 (0.96–1.60)	1.18 (0.81–1.70)	1.33 (1.01–1.76)*

Adjusted for gender, age, and educational level

*OR* odds ratio*, 95% CI 95% confidence interval,* *p < *0.05*

## Discussion

Employees with common diseases, most notably with MD, were more likely to have a low work ability and, particularly long-term, sick leave than healthy individuals. The presence of multiple common diseases additively increased the likelihood of low work ability and sick leave. Among both healthy employees and employees with common diseases, those with unfavourable psychosocial work-related factors, and to a smaller extent those with unfavourable physical work-related factors and unhealthy lifestyle-related factors, were more likely to have low work ability, and to a smaller extent sick leave.

The finding that particularly MD was strongly related to low work ability and sick leave is in accordance with the previous research (Leijten et al. 2013, 2014; Van den Heuvel et al. 2010). The previous research found that major depression, bipolar disorder, panic disorder, and post-traumatic stress disorder were associated with sick leave (De Graaf et al. 2012; Alonso et al. 2011). This might be explained by the interference of MD with regular work activities, whereas, for example, high blood pressure in employees with CVD can exist without any interference (Leijten et al. 2014).

Motivated by the rising prevalence of co-occurrence of multiple diseases, there has been a growing interest in the concept of co- and multimorbidity, as it has been associated with an increased burden on health service capacity and increased health care costs as well (Lehnert et al. 2011). This study underlines the impact of multimorbidity on adverse work-related outcomes. In line with findings from the previous research, multimorbidity increased the ORs for low work ability and sick leave (Leijten et al. 2014; Ward 2015). However, the strength of the association of more than one disease was not stronger than the sum of the risks of the underlying specific underlying diseases. Multimorbidity thus shows an additive influence rather than a synergistic influence. The previous research also did not find indications for synergistic effects between mental and physical health problems on work ability (Leijten et al. 2014).

Unfavourable psychosocial work-related factors had the strongest associations with low work ability and sick leave in both healthy employees and employees with common diseases. No consistent differences were observed between healthy employees and employees with common diseases for the associations between psychosocial work-related factors and work ability and sick leave. The finding that unfavourable psychosocial work-related factors are strongly related to low work ability and to smaller extent to sick leave is consistent with the previous research (Leijten et al. 2013; Van den Berg et al. 2009). Unfavourable psychosocial work factors seem to be a generic risk factor for low work ability and sick leave. Altogether, worksite interventions aiming at improving psychosocial work-related factors have potential to be beneficial for reducing low work ability and sick leave among all employees, regardless of their health status. This might imply that generic interventions instead of disease-specific interventions regarding psychosocial work-related factors should be developed for maintaining or increasing work ability and the prevention of sick leave.

Associations were found between unfavourable physical work-related factors and low work ability among employees in all subgroups except among employees with MD. High physical workload negatively influences physical health. Due to the nature of MD, it is likely that lower work ability among employees with MD is the consequence of reduced mental functioning instead of reduced physical functioning. Previously, high physical work demands have been associated with low work ability and have been identified as a risk factor for long-term sick leave among heterogeneous study populations (Leijten et al. 2013; Van den Berg et al. 2009; Lötters and Burdorf 2006). In this study, associations between high physical work demands and sick leave were more modest and less consistent. This might be due to different definitions of sick leave, since the previous studies showed that risk factors for long-term and short-term sick leave may differ (Van Duijvenbode et al. 2009). Based on this study, promoting favourable physical work-related factors will have a particularly positive effect on work ability and—to some extent—sick leave among healthy employees and employees with diseases that may affect physical functioning (i.e., MSD, CVD, and RD).

Concerning lifestyle-related factors, heavy smoking, insufficient physical activity, and obesity were associated with low work ability and sick leave—but differed across disease subgroups. Our finding that heavy smoking is associated with sick leave is supported by other studies (Robroek et al. 2011; Laaksonen et al. 2009). Contradictory findings have been reported on the relation between smoking and work ability (Van den Berg et al. 2009). Our results also showed less consistent associations between heavy smoking and work ability. It is conceivable that smoking contributes to the severity of particularly RD and CVD, and, therefore, has an important impact on work ability and sick leave in these particular subgroups.

In accordance with available literature, insufficient physical activity was significantly associated with low work ability and sick leave in healthy employees (Nevaper et al. 2016; Van den Berg et al. 2008) and—although not statistically significant—showed increased risks among the subgroups of MSD and RD. PA may influence the likelihood of chronicity of MSD and RD, whereas it may also be possible that the presence of disease will influence the level of PA. However, promoting physical activity among employees with RD could be beneficial, as physical inactivity among those patients is associated with adverse clinical outcomes and has been identified as an important modifiable risk factor (Troosters et al. 2013). Therefore, promoting physical activity among healthy employees and employees with MSD and RD might improve work ability and prevent sick leave and maintain work ability and prevent sick leave among healthy employees.

Obesity has been identified as a risk factor for sick leave in a large variety of occupational populations (Van Duijvenbode et al. 2009). In this study, obesity was associated with sick leave only among employees with common diseases and predominantly among employees with MSD. One study found obesity to be a risk factor for sick leave in farmers with MSD (Hartman et al. 2006). Associations between MSD and obesity have been described before, and it is postulated that both the functional and structural limitations imposed by the additional loading of the locomotor system in obesity raise stress within connective-tissue structures which is considered a risk for musculoskeletal injury (Wearing et al. 2006). Moreover, obesity was 2–3 times more prevalent in employees with common diseases than in healthy employees with low work ability or sick leave. As regards the association between obesity and low work ability, only an association was found for employees with RD. An explanation for the association between obesity among employees with RD and low work ability could be that obesity may cause sleep apnoea and obesity-associated hypoventilation, and thus will aggravate the functional impairments among workers with RD (Troosters et al. 2013; Young et al. 2004). Thus, lifestyle interventions that aim at losing weight may improve work ability among employees with RD and may reduce sick leave among employees with MSD, CVD, RD, and MD.

A strength of this study is the large study population, which consisted of 8364 paid health care employees, and the focus on multiple common diseases. Some limitations of this study should be mentioned. First, with a response level of 55%, selection bias cannot be ruled out. Although a formal non-response analysis was not possible due to privacy regulations, reasons for non-participation mentioned during interviews with key figures in each organization included lack of time, irregular work schedule, and re-organizations in the health care sector. The relatively low participation might influence the generalizability of the results. About 80% was female, which is representative of employees in this sector. However, results may differ for employees in different sectors. Therefore, the results cannot simply be generalized to other occupational groups or to other countries. Second, inherent to the cross-sectional study design, it was not possible to investigate causal relationships between the determinants, work ability, and sick leave. As a consequence, no statements can be made whether for example receiving low rewards causes low work ability in employees or that employees with low work ability experience low rewards. Third, all data were self-reported; therefore, response bias cannot be excluded. Self-reported data, however, are widely used in occupational medicine and it has shown to be a good predictor for long-term sick leave and productivity loss (Reeuwijk et al. 2014). Finally, the categories of common diseases were fairly broad and severity was not assessed; for example, both employees with a heart attack and employees with high blood pressure were combined in the CVD subgroup. The previous research showed that the severity of diseases was associated with work impairments (Detaille et al. 2009). For future research, it is recommended to have more insight into specific diagnoses.

## Conclusion

In conclusion, this study shows that common diseases, and foremost MD, were related to both low work ability and sick leave. Among both healthy employees and employees with common diseases, those with unfavourable psychosocial work-related factors, and to a smaller extent those with unfavourable physical work-related factors and unhealthy lifestyle-related factors, were more likely to have low work ability, and to a smaller extent sick leave. To maintain or improve work ability and prevent sick leave, interventions that promote a healthy psychosocial work environment are needed.
